# Immobilization of Pb and Zn in Contaminated Soil Using Alumina–Silica Nano-Amendments Synthesized from Coal Fly Ash

**DOI:** 10.3390/ijerph192316204

**Published:** 2022-12-03

**Authors:** Chang Lei, Hao Huang, Haoxin Ye, Zhiping Fu, Peipei Peng, Shaoqing Zhang, Laishou Long

**Affiliations:** School of Chemistry and Civil Engineering, Shaoguan University, Shaoguan 512005, China

**Keywords:** alumina–silica nano-amendments, coal fly ash, heavy metals, soil remediation, immobilization

## Abstract

To apply coal fly ash to the remediation of heavy-metal-contaminated soil, an alumina–silica nano-amendment (ASNA) was synthesized from coal fly ash and was used for the immobilization of lead and zinc in contaminated soil. The investigation on the synthesis of the ASNA shows that the ASNA can be obtained under a roasting temperature of 700 °C, a ratio of alkali to coal fly ash of 1.2:1, and a molar ratio of silicon to aluminum of 1:1. The ASNA could increase the soil pH and cation exchange capacity (CEC) and decrease the bioavailability of Pb and Zn. When the ASNA addition increased from 0 to 2%, the bioavailability (extracted by CaCl_2_) of Pb and Zn decreased by 47% and 72%, respectively. Moreover, the addition of the ASNA facilitated the transformation of Pb from a reducible fraction to oxidizable and residual fractions and Zn from an exchangeable fraction to a residual fraction. The correlation analysis and cluster analysis verify that the ASNA modulates the chemical speciation of heavy metals by increasing the soil’s CEC and pH, thereby immobilizing heavy metals. It is expected that this study can provide a new method for the remediation of Pb- and Zn-contaminated soil.

## 1. Introduction

The continuous expansion of the urban scale and the rapid increase in industrial activities have caused a large amount of harmful heavy metals to enter the soil through various channels, resulting in serious environmental pollution [1,2]. Since heavy metals are not degradable, their potential toxicity and high persistence not only have a serious impact on the yield and quality of crops but also endanger human health through the food chain [3,4].

In situ immobilization is a common method for the remediation of heavy metal contamination in farmland soil. The main principle is to reduce the harmfulness of heavy metals by adding amendments [5]. After adding amendments, the chemical speciation of heavy metals that easily migrates is transformed into the chemical speciation that plants do not easily absorb through a series of reactions such as adsorption, precipitation, complexation, and ion exchange between the soil and heavy metal ions [6]. Although this method has the advantages of simple operation, low cost, less engineering, and less damage to the original function of soil, since heavy metals cannot be completely removed from the soil, the remediation agent needs to have good performance to ensure a good remediation effect [7].

In the current in situ immobilization technology for heavy metals in farmland soil, the commonly used amendments mainly include calcareous materials [8], phosphorus-containing materials [9], carbon materials [10], clay minerals [11], organic fertilizers [12], and agricultural wastes [13]. These traditional amendments have been criticized for their low remediation efficiency, high dosage required, and significant side effects on farmland soil [14].

Alumina–silica nanomaterials, as nanoscale particles, have a large specific surface area due to their silicon–oxygen tetrahedral structure, so they have a high cation exchange capacity and high adsorption capacity. Because of their unique structural characteristics and properties, they are often used as adsorbents [15,16]. Many studies have shown that applying alumina–silica nano-amendments to polluted soil can significantly reduce the bioavailability of heavy metals in soil [17,18,19].

Coal fly ash is the solid waste of modern industries such as power plants. With the development of the power industry, the discharge of fly ash from coal-fired power plants has increased year by year [20,21], and various materials are prepared to comprehensively use it. The preparation of coal fly ash as a soil-remediation agent for heavy-metal-contaminated soil is widely used. Chemical agents are often used to modify coal fly ash to improve its adsorption performance for heavy metals. However, these common modification methods have a poor modification effect, low adsorption efficiency, and insufficient stability after the adsorption of heavy metals. Alumina–silica nano-amendments are a new type of nanomaterial. Due to their nanostructures and high specific surface area, they have a great ability to adsorb and immobilize heavy metals. Alumina–silica nano-amendments synthesized by coal fly ash can not only effectively solve the low adsorption capacity of modified fly ash for heavy metals but also reduce the synthesis cost of alumina–silica nano-amendments.

At present, there are some methods for preparing alumina–silica nano-amendments from coal fly ash, including the traditional hydrothermal synthesis method [22], two-step hydrothermal synthesis method [23], microwave-assisted method [24], seed method [25], and alkali fusion method [26]. However, alumina–silica nano-amendments synthesized by different methods have different performances and great cost differences. To obtain alumina–silica nano-amendments with better performance at a low cost, it is necessary to study the influence of basic condition parameters on alumina–silica nano-amendments’ synthesis. Moreover, although alumina–silica nano-amendments have been applied to treat heavy metal pollution in soil, the immobilization mechanism of alumina–silica nano-amendments for heavy metals has not been studied in detail. It is generally believed that alumina–silica nano-amendments can affect the adsorption performance by affecting soil properties such as pH, CEC, and OM. However, the relationship between the immobilization effect of heavy metals and the physical and chemical properties of soil is still not well understood. Therefore, the immobilization mechanism of alumina–silica nano-amendments for heavy metals remains to be further studied.

In this study, coal fly ash was used as a raw material to prepare an alumina–silica nano-amendment with excellent performance by exploring the condition parameters of the alkali fusion–hydrothermal synthesis process. Furthermore, the alumina–silica nano-amendments were used to immobilize the heavy metals lead and zinc in soil. This study is expected to provide a method for the remediation and control of heavy metal pollution in farmland soil.

## 2. Materials and Methods

### 2.1. Materials

The coal fly ash used in the synthesis experiment of an alumina–silica nano-amendment (ASNA) was obtained from a power plant in Guangdong, China. The reagents used in the experiment included sodium hydroxide, sodium aluminate, and sodium silicate. All chemical reagents were of analytical grade.

The soil used in the immobilization experiment of heavy metals was collected from the soil surface (0–17 cm) of contaminated paddy fields (113.65 °E, 25.11 °N) around the Fangkou lead–zinc mine in Renhua, Guangdong, China. In this region, the heavy metal content in the contaminated soil is associated with wastewater produced by the mining industry [1]. After removing stones and sundries, the soil was mixed, air-dried, and passed through a 100-mesh sieve. The pH of the soil was 6.16. The organic matter (OM) content and the cation exchange capacity (CEC) were 1.81% and 21.3 cmol/kg, respectively. The contents of Pb and Zn were 1891.7 and 985.1 mg/kg, respectively, which were higher than the risk screening values of 100 and 200 mg/kg of lead and zinc in the soil environmental quality risk control standard for soil contamination of agricultural land [27]. This paper focuses on the influence of ASNA on lead and zinc in the soil.

### 2.2. The Synthesis Experiment of an ASNA Using Coal Fly Ash

The alkali fusion hydrothermal synthesis method was adopted to synthesize ASNA. A mass of coal fly ash was weighed as the raw material, and sodium hydroxide was added at a set ratio as the alkali source. Sodium silicate or sodium aluminate was added as the silicon source or aluminum source. After being mixed and ground, they were put into a porcelain crucible and roasted at a high temperature in a muffle furnace for 2 h to destroy the quartz and mullite in the coal fly ash. Then, the calcined clinker was cooled, ground, water was added, and it was aged at room temperature for 12 h. After that, it was added to the reaction kettle and crystallized at a certain temperature for 8 h. The ASNA was obtained after filtering, washing, and drying.

### 2.3. The Immobilization Experiment of Heavy Metals in the Soil

We accurately weighed 4 parts of 500 g air-dried and sieved soil and added 0, 2.5, 10, and 25 g of ASNA, respectively. Distilled water was added to maintain the water at 70% of the field capacity of the soil. After standing for one year, soil pH, OM, CEC, and chemical speciation and bioavailability of Pb and Zn were determined.

### 2.4. Analytical Methods

The phase of the experimental samples was determined by Japan Rigaku D/max-2000 X-ray diffractometer. The soil pH was measured by the following method. The soil and deionized water were thoroughly mixed in a ratio of 1:2.5, then placed in a water bath constant temperature oscillator and shaken at room temperature for 3 h. After that, it was taken out and measured with a PHS-3C pH meter. The CEC was determined by the barium chloride–sulfuric acid forced exchange method [28]. The organic matter in the soil was determined by the potassium dichromate–sulfuric acid method [29].

The heavy metal content in soil was determined by the following method. We accurately weighed 0.1 g of the dried soil (dry at 105 °C for 8 h) that passed through a 100-mesh sieve and put it in a high-pressure digestion tank. We added 2 mL HCl, 2 mL HNO_3_, and 1 mL HF and placed it in an oven. After heating at 170 °C for 8 h, the digestion tank was taken out and cooled to room temperature. Then, it was put on an electric hot plate and heated to drive off acid. After transferring the solution to a volumetric flask, the concentration of lead and zinc was measured by a WFX-110A flame/graphite furnace atomic absorption spectrophotometer.

The chemical speciation of the soil’s heavy metals was determined by the modified BCR sequential extraction method. In this method, the soil’s heavy metals in the exchangeable (EX), reducible (RE), and oxidizable (OX) fractions were extracted sequentially by 0.11 mol/L HOAc, 0.1 mol/L NH_2_OH·HCl, and 8.8 mol/L H_2_O_2_, followed by 1.0 mol/L NH_4_Oac, respectively. After extraction, the heavy metals remaining in the soil were the residual fraction (RES) [30].

Three methods were used to determine the bioavailability of heavy metals in the soil, namely, the CaCl_2_ extraction method, the DTPA extraction method, and the toxicity characteristic leaching procedure (TCLP). The bioavailability extracted by CaCl_2_ (B-CA) was measured by extracting heavy metals in soil with 0.01 mol/L CaCl_2_ [31]. The bioavailability extracted by DTPA (B-DTPA) was measured by extracting heavy metals in soil with the solution of 0.005 mol/L DTPA, 0.01 mol/L CaCl_2_, and 0.01 mol/L TEA [32]. The bioavailability obtained from the TCLP method (B-TCLP) was measured by extracting heavy metals in soil with a solution of glacial acetic acid (pH 2.88 ± 0.05) [33].

### 2.5. Quality Control and Data Analysis

To ensure the accuracy of the data, all experiments were performed in triplicate. The average values were used as the results. The standard deviations were estimated for the immobilization experiment. Data were processed by Microsoft office, Jade 6, and Origin 8. The correlation analysis was carried out using SPSS 17. The cluster analysis was performed with R Studio.

## 3. Results and Discussion

### 3.1. Characteristics of Coal Fly Ash

The chemical composition of coal fly ash was analyzed by XRF. The results are shown in Table 1. The main components of the coal fly ash are SiO_2_ and Al_2_O_3_, which account for 88.32% of the total components. The remaining components include small amounts of CaO, Fe_2_O_3_, and K_2_O. Since there are few impurities in this part, pickling and removing impurities from the fly ash can be omitted.

**Figure 1:**
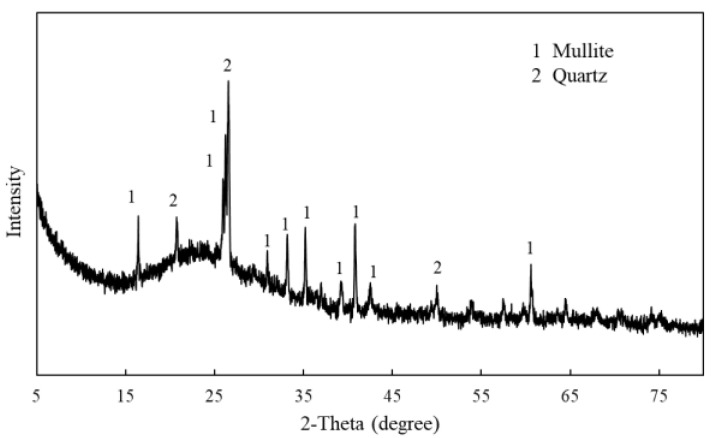
The XRD pattern of coal fly ash.

To further confirm the phase composition in the coal fly ash, an XRD analysis was carried out. As shown in Figure 1, the main phases of the coal fly ash are quartz (SiO_2_) and mullite (Al_6_Si_2_O_13_), which is consistent with the analysis of the chemical composition of the coal fly ash above. Since the composition of the coal fly ash and alumina–silica nanomaterial are consistent, using the coal fly ash as a raw material can effectively synthesize an alumina–silica nanomaterial. The molar ratio of silicon to aluminum in the coal fly ash is 1.39:1, close to that of the alumina–silica nanomaterial. Therefore, in terms of composition, it is feasible to synthesize an alumina–silica nanomaterial from coal fly ash.

### 3.2. The Synthesis of the ASNA Using Coal Fly Ash

**Figure 2:**
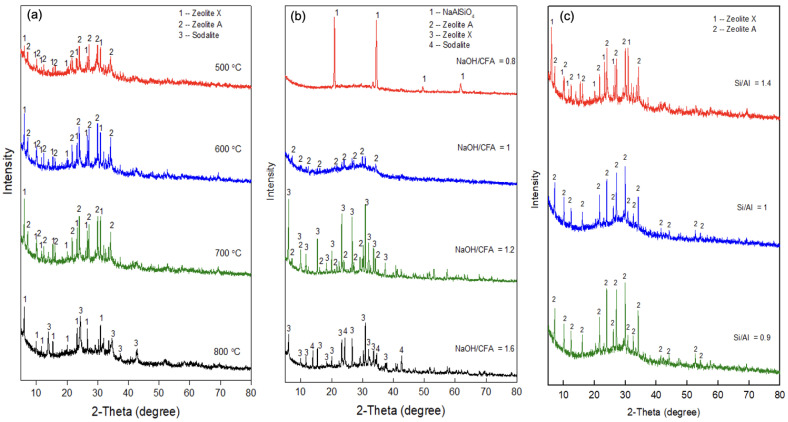
XRD patterns of ASNA synthesized under different roasting temperatures (**a**), ratios of alkali (NaOH) to coal fly ash (**b**), and molar ratios of silicon to aluminum (**c**).

Figure 2 shows the phase changes of the ASNA synthesized under different conditions. As shown in Figure 2a, ASNA can be synthesized at 500–700 °C. Zeolite A and zeolite X were the main components. The ASNA obtained at 600 °C and 700 °C has higher peaks than that obtained at 500 °C. This may be because the higher temperature was more conducive to converting components such as quartz and mullite in the coal fly ash into the active silica–aluminum phase [34], and thus, more ASNA was synthesized. However, when the temperature reached 800 °C, more sodalite began to appear, indicating that the temperature was too high to be favorable for the formation of ASNA. When the temperature was 600–700 °C, a well-crystallized ASNA could be obtained, so the roasting temperature is the best within this range.

As shown in Figure 2b, when the ratio of alkali to coal fly ash was 0.8, the main component of the obtained product was sodium aluminum silicate. This may be because when the amount of alkali is low, the quartz and mullite crystal phase in the coal fly ash is destroyed to form sodium silicate aluminate, but its activity is relatively weak, and an ASNA cannot be formed during the crystallization process [35].

With the increase in the alkali addition amount, ASNA was produced in the form of zeolite A and zeolite X. When the ratio of alkali to coal fly ash reached 1.2, high peaks of zeolite A and zeolite X could be obtained. However, when the ratio of alkali to coal fly ash was increased to 1.6, the peak of sodalite crystal was accompanied by the peaks of zeolite A and zeolite X. This may be because when the amount of alkali is too great, the alkalinity in the system during the crystallization reaction is strong, which reduces the degree of polymerization of silicate ions and forms sodalite [36]. Therefore, the alkali addition amount can neither be too high nor too low. The ratio of alkali to coal fly ash of 1.2 is favorable for synthesizing ASNA.

The XRD pattern of ASNA synthesized under different molar ratios of silicon to aluminum is shown in Figure 2c. When the molar ratio of silicon to aluminum was 1.4, the main components of the ASNA were zeolite X and zeolite A, indicating that different zeolite crystals easily formed at the same time when the molar ratio of silicon to aluminum was high. When the molar ratio of silicon to aluminum was 1 or 0.9, the component of the ASNA was mainly zeolite A, indicating that the obtained ASNA was crystalline. Thus, the ASNA obtained from coal fly ash under a roasting temperature of 700 °C, a ratio of alkali to coal fly ash of 1.2:1, and a molar ratio of silicon to aluminum of 1:1 was used as a soil amendment for the immobilization of heavy metals in soil. Although the roasting temperature required for the preparation of the ASNA is high, the cost will be greatly reduced compared with nanomaterials made from raw materials such as sodium silicate and sodium aluminate because the raw material used in this method is coal fly ash.

### 3.3. The Effect of the ASNA on the Immobilization of Pb and Zn in Contaminated Soil

#### 3.3.1. The Changes in the Soil’s Physical and Chemical Properties

**Figure 3:**
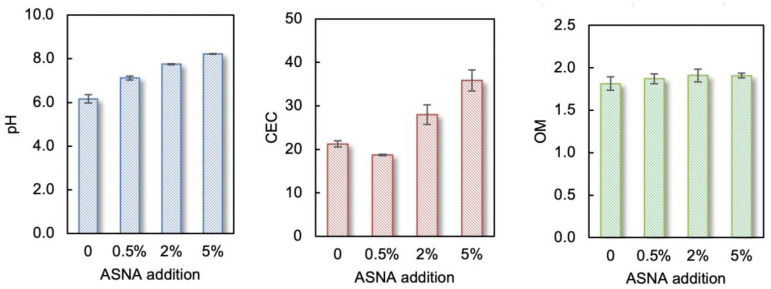
Effects of ASNA on soil pH, CEC, and OM.

The effect of the ASNA on the soil’s physical and chemical properties was investigated by adding different amounts of ASNA into the soil. It can be seen in Figure 3 that the addition amount of ASNA has little effect on the OM in soils. After adding different amounts of ASNA, the maximum and minimum OM contents in the soils were 1.98% and 1.81%, respectively. The variation range is between 0.1% and 0.2%. The pH of the soil increased with the increase in the addition amount of the ASNA. Larger addition amounts of ASNA caused higher pH values. This may be because the ASNA provided adsorption sites for H+ when added to acidic soil, thereby increasing the pH value of the soil [37]. In addition, the porous structure of ASNA can increase its adsorption of heavy metals to a certain extent, thereby reducing the bioavailability of heavy metals.

The CEC of soil is an important indicator of soil fertilization and buffering capabilities [38]. After adding 0.5% of the ASNA to the soil, the CEC of the soil changed a little. However, when the addition amount of the ASNA reached 2% and 5%, the CEC of the soils showed an upward trend. A larger amount of the ASNA led to a higher CEC. This may be because the ASNA provides the hydroxy-aluminum component, which is confirmed to be one of the sources of a large portion of the CEC in the soil [38]. The increase in CEC can reduce the exchange of heavy metals such as Pb and Zn in the paddy fields, affecting their bioavailability [39].

#### 3.3.2. The Changes in the Bioavailability of Pb and Zn in the Soil

**Figure 4:**
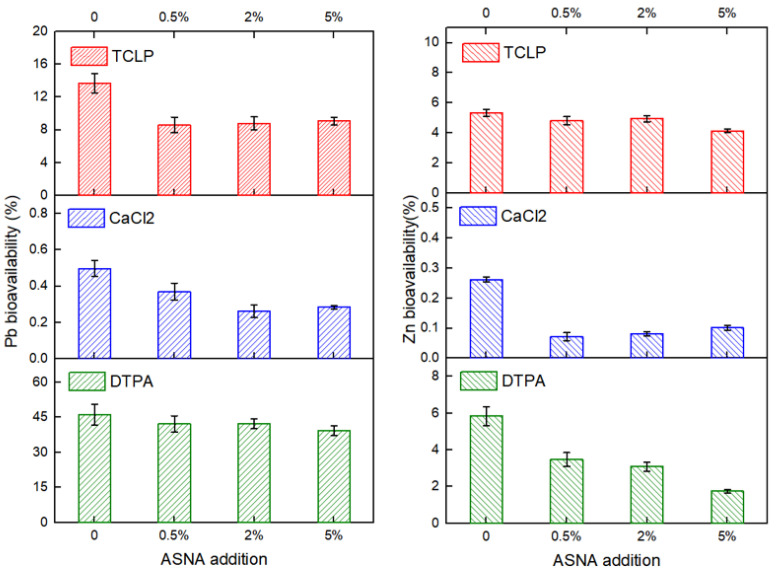
The effect of ASNA on the bioavailability of Pb and Zn in soil.

To accurately reveal the degree of harm of heavy metals in soil, three different methods, including the CaCl_2_ extraction method, DTPA extraction method, and toxicity characteristic leaching procedure (TCLP), were used to determine the bioavailability of heavy metals in the soil. Figure 4 shows the changes in the bioavailability of Pb and Zn in the soil under different addition amounts of the ASNA. The bioavailability of Pb and Zn both showed a downward trend with the increase in the addition amount of the ASNA in the soil. When the addition amount of the ASNA increased from 0 to 0.5%, the B-TCLP of Pb decreased greatly, reaching 37%. When the ASNA addition increased from 0 to 2%, the B-CA of Pb decreased the most, reaching 47%. Additionally, with the increase in the addition amounts of the ASNA, the B-DTPA of Pb also decreased but by a smaller margin, with only a 15% decrease at the ASNA addition amount of 5%. This may be due to the higher B-DTPA of Pb than B-TCLP and B-CA.

In contrast to lead, the B-DTPA of Zn is relatively low. Moreover, the range of the decrease in the B-DTPA of Zn is larger than that of Pb with the increase in the addition amount of the ASNA. The range of decrease could reach 70% when the addition amount of the ASNA was 5%. However, the B-TCLP of Zn decreased slightly with the increase in the addition amount of the ASNA. The range of decrease was only 22% with the ASNA addition amount of 5%. Similar to Pb, the B-CA of Zn decreased the most when the ASNA addition amount increased from 0 to 0.5%, reaching 72%. Feng et al. reported that CaCl_2_ extraction methods were suitable for exchangeable metals [32]. Thus, it can be deduced that adding the ASNA benefits the immobilization of Pb and Zn in the soil. The results show that the ASNA has great application potential in treating heavy-metal-contaminated soil.

However, due to the different binding forms of the ASNA and heavy metals, the effect of the ASNA on the bioavailability obtained by different extractions is different [32]. To further clarify the effect of the ASNA on the immobilization of heavy metals in soil, it is necessary to study the effect of the ASNA on the chemical speciation of heavy metals.

#### 3.3.3. The Changes in the Chemical Speciation

**Figure 5:**
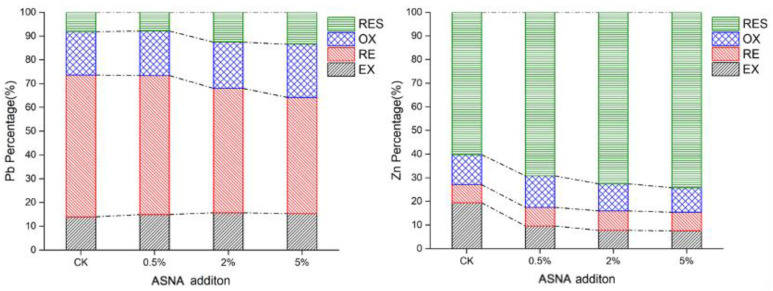
The chemical speciation of Pb and Zn in soils with different addition amounts of ASNA.

Figure 5 shows the chemical speciation of lead and zinc in the soil with different addition amounts of the ASNA. Lead was mainly present in RE, which can reach 59.7% without the addition of the ASNA. However, after the addition of the ASNA, the proportion of RE gradually decreases. When the addition of the ASNA is 5%, its proportion decreases to 48.9%, a decrease of 18.1%. Furthermore, the proportion of OX and RES of lead increases gradually with the addition amount of the ASNA. When the addition amount of the ASNA is 5%, the proportion of OX and RES of lead reaches the maximum, which is 22.4% and 13.4%, respectively. The addition of the ASNA is beneficial to the transformation of lead from RE to OX and RES in the soil, which is consistent with previous research [3]. Since the order of mobility of speciation in the soil is EX > RE > OX > RES [4], the addition of the ASNA can effectively reduce the mobility of heavy metals, which is consistent with the above results on the ASNA’s effect on the bioavailability of lead.

Unlike lead, in the absence of the ASNA, zinc is mainly present in the RES, accounting for 60.2%. After adding the ASNA, the RES of zinc is further improved. When the addition amount of the ASNA is 5%, the proportion of the RES of zinc rises to 69.2%. Moreover, among the other three fractions of zinc, the EX is most affected by the ASNA. When the addition amount of the ASNA is 5%, the proportion of the EX of zinc decreases to 7.5%, up to 61.5% lower than that in the soil without the ASNA’s addition. These results indicated that the ASNA was beneficial to the transfer of zinc from EX to RES in the soil, thus, reducing the mobility of zinc in the soil.

### 3.4. Immobilization Mechanism

The addition of ASNA changes the physical and chemical properties of soil, such as the pH and CEC. Furthermore, these physical and chemical properties also affect the change in the speciation and bioavailability of heavy metals. To explore the immobilization mechanism, a correlation analysis was carried out on these parameters and the speciation of Pb and Zn in the soil. The results are shown in Table 2 and Table 3.

As shown in Table 2, the addition amount of the ASNA was positively correlated with CEC (significant) and OX-Pb (extremely significant) and negatively correlated with RE-Pb (significant). This confirmed the previous conclusion that the addition of the ASNA not only promoted the increase in CEC but also accompanied the decrease in RE-Pb and the increase in OX-Pb. Additionally, there is a significant negative correlation between the pH and the B-DTPA and B-CA of Pb, indicating that the increase in pH was accompanied by the decrease in B-DTPA and B-CA of Pb. This result is consistent with the report that the decrease in the B-CA of heavy metals in the amended soils can be attributed partly to the significant increase in the soil’s pH [6].

Moreover, there is a significant negative correlation between the CEC and the RE-Pb, which indicates that the effect of the ASNA on RE is probably due to increasing the CEC of the soil. The increase in CEC improved the adsorption of Pb ions by the soil and reduced the possibility of Pb ions being bound by reducing substances. Cui et al. suggested that soils with low CEC showed a decreased ability to detoxify heavy metals, resulting in a substantial decrease in plant biomass [39]. This finding verified the above result. In addition, the RE and the RES show a significant negative correlation, indicating that under the influence of the ASNA, the chemical speciation of Pb changed from RE to RES.

According to the above discussion, it can be inferred that the immobilization of lead in soil by the ASNA undergoes the following process. First, the addition of the ASNA significantly increased the CEC in the soil. Then, under high CEC, the RE of Pb was significantly reduced. This part of reduced Pb was released from the RE and combined with the ASNA. Due to the strong binding ability of the ASNA and lead, this part of lead cannot be extracted by the first three extractants in BCR, so it becomes the RES. Through this process, the ASNA transforms Pb from the highly migratory RE to the more stable RES.

**Figure 6:**
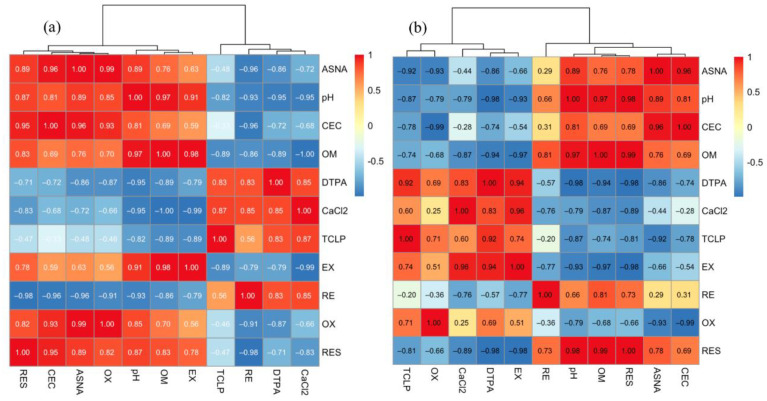
Cluster analysis of the correlation coefficients between soil physical and chemical properties and the chemical speciation and bioavailability of Pb (**a**) and Zn (**b**).

To verify our inference, a cluster analysis was conducted on the correlation coefficients between the soil properties and the Pb’s chemical speciation and bioavailability. The results are shown in Figure 6a. The RE obtained from BCR and the B-CA, B-DTPA, and B-TCLP clustered into one group. Although the bioavailability extracted by the three extraction methods is different, they are also consistent to a certain extent, which can indirectly reflect the change in the bioavailability of heavy metals in soil. Additionally, the RE was more closely related to bioavailability. This may be because most Pb in the soil was present in the form of oxide binding, and most of the Pb extracted by these three extractants was also in this form. Moreover, EX, OM, and pH were also clustered into one group; that is, the increase in OM and pH was accompanied by the increase in EX. This may be because the EX of lead mainly exists under the action of pH and the combination of OM, which directly affect the adsorption and exchange of heavy metal Pb, thus affecting the EX of Pb. It is worth noting that the addition amount of the ASNA, CEC, OX, and RES are also clustered into one group, which confirms that the addition of the ASNA may lead to the increase in CEC, thus increasing the RES, which is consistent with the results mentioned above.

Table 3 shows the correlation analysis of Zn’s chemical speciation, bioavailability, ASNA addition, and soil physicochemical properties. Similar to lead, the pH is also significantly negatively correlated with the B-DTPA of Zn, indicating that the increase in the pH is conducive to reducing the bioavailability of Zn. OM was significantly negatively correlated with the EX and RES of Zn. This indicates that the increase in OM can decrease the EX and improve the RES. Therefore, increasing OM in soil may be an effective method to reduce the migration of Zn. Li et al. also suggested that increasing soil OM can increase the content of organ-bound metals in soil and reduce a soil’s heavy metals availability [4].

Figure 6b shows the cluster analysis of the correlation coefficients between soil properties and Zn chemical speciation and bioavailability. The EX-Zn and the B-DTPA and B-TCLP of Zn were clustered into one group, indicating that the B-DTPA and B-TCLP of Zn are consistent with EX, and both can reflect the mobility of Zn in soil. However, the OX-Zn and the B-TCLP of Zn were clustered into one group, indicating that the B-TCLP of Zn could better reflect OX. The OM, pH, and RES-Zn were clustered into one group, indicating that the increase in pH and OM leads to increased RES. This is because the increase in pH and OM will lead to heavy metal precipitation or combination with organic matter to form chemical speciation that is not easy to migrate [4]. In addition, the addition amount of the ASNA and CEC were clustered into one group. This was consistent with the previous study; that is, the addition amount of the ASNA was closely related to the change in CEC. These results verify that the ASNA modulates the chemical speciation of heavy metals by increasing the soil’s CEC and pH, thereby altering the migration of heavy metals and immobilizing heavy metals.

## 4. Conclusions

The alkali fusion hydrothermal synthesis method was adopted to synthesize an ASNA by coal fly ash. The crystalline ASNA can be obtained under a roasting temperature of 700 °C, a ratio of alkali to coal fly ash of 1.2:1, and a molar ratio of silicon to aluminum of 1:1. The ASNA has great application potential in the treatment of heavy metal contaminated soil.

The ASNA was used for the remediation of heavy-metal-contaminated soil. The ASNA did not affect the soil’s OM but increased the soil’s pH and CEC. The ASNA could reduce the bioavailability of Pb and Zn extracted by three different methods, including the CaCl_2_ extraction method, the DTPA extraction method, and the TCLP method. When the ASNA addition increased from 0 to 2%, the B-CA of Pb and Zn decreased by 47% and 72%, respectively. The addition of the ASNA contributed to the immobilization of Pb and Zn in the soil. The change in chemical speciation shows that adding the ASNA benefits the transformation of lead from RE to OX and RES in the soil. When the addition amount of the ASNA was 5%, the proportion of OX and RES of lead reached the maximum, which was 22.4% and 13.4%, respectively. Additionally, the ASNA is beneficial to the transfer of zinc from EX to RES in the soil. When the addition amount of the ASNA was 5%, the proportion of the RES of zinc rose to 69.2%.

The correlation analysis shows a significant negative correlation between the pH and the bioavailability of Pb and Zn. The cluster analysis shows that the addition amount of the ASNA and CEC were clustered into one group. The result shows that the ASNA modulates the chemical speciation of heavy metals by increasing the soil’s CEC and pH, thereby altering the migration of heavy metals and immobilizing heavy metals in the soil.

## Figures and Tables

**Table 1:** Chemical composition of coal fly ash.

Components	SiO_2_	Al_2_O_3_	CaO	Fe_2_O_3_	K_2_O	TiO_2_	MgO	Na_2_O	SO_3_	Others
Wt%	54.9	33.5	4.15	2.48	1.98	1.09	0.748	0.465	0.405	0.362

**Table 2:** Correlation analysis of Pb chemical speciation, bioavailability, ASNA addition, and soil physicochemical properties.

	ASNA	pH	CEC	OM	DTPA	CaCl2	TCLP	EX	RE	OX	RES
ASNA	1										
pH	0.885	1									
CEC	0.964 *	0.814	1								
OM	0.755	0.971 *	0.694	1							
DTPA	−0.863	−0.950 *	−0.720	−0.886	1						
CaCl2	−0.724	−0.954 *	−0.679	−0.997 **	0.847	1					
TCLP	−0.484	−0.817	−0.329	−0.886	0.833	0.872	1				
EX	0.629	0.908	0.588	0.981 *	−0.788	−0.992 **	−0.886	1			
RE	−0.956 *	−0.934	−0.961 *	−0.865	0.831	0.854	0.562	−0.787	1		
OX	0.992 **	0.851	0.934	0.702	−0.866	−0.661	−0.459	0.558	−0.912	1	
RES	0.889	0.871	0.946	0.826	−0.710	−0.831	−0.473	0.775	−0.979 *	0.824	1

** The correlation was significant at 0.01 level (two-tailed). * The correlation was significant at 0.05 level (two-tailed).

**Table 3:** Correlation analysis of Zn chemical speciation, bioavailability, ASNA addition, and soil physicochemical properties.

	ASNA	pH	CEC	OM	DTPA	CaCl2	TCLP	EX	RE	OX	RES
ASNA	1										
pH	0.885	1									
CEC	0.964 *	0.814	1								
OM	0.755	0.971 *	0.694	1							
DTPA	−0.859	−0.980 *	−0.738	−0.938	1						
CaCl2	−0.438	−0.789	−0.284	−0.869	0.834	1					
TCLP	−0.916	−0.865	−0.777	−0.736	0.919	0.602	1				
EX	−0.660	−0.929	−0.540	−0.969 *	0.942	0.960 *	0.741	1			
RE	0.291	0.657	0.314	0.813	−0.569	−0.755	−0.204	−0.772	1		
OX	−0.930	−0.788	−0.993 **	−0.684	0.691	0.250	0.705	0.511	−0.359	1	
RES	0.782	0.981 *	0.686	0.989 *	−0.976 *	−0.892	−0.812	−0.983 *	0.734	−0.659	1

** The correlation was significant at 0.01 level (two-tailed). * The correlation was significant at 0.05 level (two-tailed).
